# Drosophila *Hsp67Bc* hot-spot variants alter muscle structure and function

**DOI:** 10.1007/s00018-018-2875-z

**Published:** 2018-07-21

**Authors:** Jadwiga Jabłońska, Magda Dubińska-Magiera, Teresa Jagla, Krzysztof Jagla, Małgorzata Daczewska

**Affiliations:** 1 Department of Animal Developmental Biology, Institute of Experimental Biology, University of Wroclaw, Sienkiewicza 21, 50-335 Wrocław, Poland; 2 GReD, INSERM U1103, CNRS, UMR6293, University of Clermont Auvergne, 28, Place Henri Dunant, 63000 Clermont-Ferrand, France

**Keywords:** Muscle, *Drosophila*, Hsp67Bc, Mutants, Development, sHSP, Protein aggregates, NMJ

## Abstract

The *Drosophila Hsp67Bc* gene encodes a protein belonging to the small heat-shock protein (sHSP) family, identified as the nearest functional ortholog of human HSPB8. The most prominent activity of sHSPs is preventing the irreversible aggregation of various non-native polypeptides. Moreover, they are involved in processes such as development, aging, maintenance of the cytoskeletal architecture and autophagy. In larval muscles Hsp67Bc localizes to the Z- and A-bands, which suggests its role as part of the conserved chaperone complex required for Z-disk maintenance. In addition, Hsp67Bc is present at neuromuscular junctions (NMJs), which implies its involvement in the maintenance of NMJ structure. Here, we report the effects of muscle-target overexpression of *Drosophila Hsp67Bc* hot-spot variants Hsp67BcR126E and Hsp67BcR126N mimicking pathogenic variants of human HSPB8. Depending on the substitutions, we observed a different impact on muscle structure and performance. Expression of Hsp67BcR126E affects larval motility, which may be caused by impairment of mitochondrial respiratory function and/or by NMJ abnormalities manifested by a decrease in the number of synaptic boutons. In contrast, Hsp67BcR126N appears to be an aggregate-prone variant, as reflected in excessive accumulation of mutant proteins and the formation of large aggregates with a lesser impact on muscle structure and performance compared to the Hsp67BcR126E variant.

## Introduction

Small heat-shock proteins (sHSPs; HSPB) constitute an evolutionarily conserved group of low molecular weight (ranging from 10 to 40 kDa) ubiquitous proteins present in almost all organisms [38]. The number of genes coding for sHSPs varies among different organisms. For example, ten sHSPs have been identified in humans and mice [51]. sHSPs are characterized by the presence of a highly conserved alpha-crystallin domain (ACD) containing 80–100 residues located close to the C-terminus, which has been shown to be essential for the solubilization and chaperone activity. The sHSPs are involved in the response to stress conditions such as heat or oxidative stress. Their most prominent activity is preventing, in an ATP-independent manner, the irreversible aggregation of various non-native proteins, and the formation of potentially pathogenic aggregates. Through these activities, sHSPs are involved in development, aging, regulation of apoptosis, maintenance of the cytoskeleton architecture, and autophagy [15, 51].

The sHSPs’ mode of action in cells strictly depends on their oligomerization and phosphorylation states. The ACD is involved in dimer formation, whereas the N-terminus is found to stabilize highly organized oligomers. Of note, sHSPs have an ability to form heterooligomeric structures. In the inactive state, most sHSPs form large oligomers, assembled from dimers. Dissociation of both monomers and dimers from the oligomers is required for sHSPs’ activity [17]. This is crucial for binding unfolded peptides to protect them against precipitation in the form of potentially pathogenic aggregates. During the stress response, sHSPs form the first line of defense against protein aggregation. The ACD domain contains several sites involved in the interaction of sHSPs with partially unfolded or structurally affected protein targets.

The sHSPs also possess specific binding sites for proteins, which are components of the protein quality control (PQC) system. This facilitates cooperation of sHSPs with other chaperone systems. Some of the sHSP family members interact with the partner protein Bcl-2-associated athanogene-3 (BAG3). BAG3 is a 61-kDa protein characterized by the presence of multiple protein–protein interaction motifs. It connects sHSPs with HSP70, involved in protein refolding, or with Hsc70 (heat-shock cognate protein), making links with autophagy control and proteasomal degradation [11, 12, 16]. Interestingly, HSPB8, which modulates autophagy-mediated protein degradation via the eIF2 pathway, also acts in a complex with BAG3 [10]. This cooperation, also found between functional *Drosophila* counterparts of HSPB8 and BAG3, the Hsp67Bc and Starvin proteins, facilitates the clearance of misfolded aggregate-prone proteins [7, 10]. Notably, BAG3 through regulation of Hsp70/Hsc70 chaperone activity plays a role in maintenance of the myofibrillar structure. Like its human ortholog, *Drosophila* Hsp67Bc localizes to the Z-band in muscle tissue, suggesting that it could contribute to the conserved chaperone complex required for Z-disk maintenance [2, 8, 10, 21, 43].

In humans, mutations in genes coding for some of the sHSPs are involved in different pathologies such as distal hereditary motor neuropathy (dHMN), desmin-related myopathy, Charcot–Marie–Tooth disease (CMT) and neurodegenerative disorders including Alzheimer’s and Parkinson’s disease, and polyglutamine repeat disorders (e.g. Huntington’s disease and spinocerebellar ataxia type 3) [3]. Although mutations underlying the pathogenesis of these diseases concern a variety of genes, they have a common denominator—aggregation of mutated proteins. In the case of sHSPs, the disease-associated mutations often concern hot-spot residues (the most relevant protein–protein interaction residues) located in the ACD. Mutations in the β5–β7 strands of the ACD may disturb the structure of sHSP monomers and inter-monomer interactions, as well as the binding of sHSPs to protein substrates [22, 28, 44].

For example, mutation R120G of a hot-spot residue in *HSPB5* leads to desmin-related myopathy (DRM), which is inherited in an autosomal-dominant manner and causes a loss of the chaperone activity of HSPB5 in vitro. It has been reported that the R120G mutation enhances the binding capacity of HSPB5 to desmin and decreases its dissociation constant [52].

Regarding HSPB8, K141E hot-spot mutation causes autosomal-dominant distal hereditary motor neuropathy (distal HMN type IIA, OMIM 158590) [27], whereas its variant (K141N) causes Charcot–Marie–Tooth disease type 2L (CMT2L, OMIM 608673) [47]. Moreover, the HSPB8-binding partner HSPB1 is also implicated in analogical disorders and its S135F mutation causes dHMN and Charcot–Marie–Tooth disease type 2F (CMT2F, OMIM 606595) [18]. These related disorders are inherited defects of motor neurons characterized by loss of muscle tissue and touch sensation. It has been proposed [14] that disruption of mutual interaction between members of the sHSP family could play a role in the pathogenesis of all inherited motor neuron diseases (MNDs).

In mammalian cells, both HSPB8 mutant forms (K141E and K141N) show increased binding to themselves as well as to other HSPBs including HSPB5 and HSPB1. Similarly, S135F HSPB1 mutation causes increased interaction with HSPB8. These observations support a view of common HSPB-involving MND mechanisms [19]. Abnormalities in mutant protein interactions also involve alterations of their isoelectric point (pI) compared to wild-type polypeptides (e.g. pI of HSPB8 wt is 4.7 whereas pIs of HSPB8^K141E^ and HSPB8^K141N^ are 4.2 and 4.3, respectively) [5, 19]. Single-point mutations may thus contribute to dramatic changes in protein architecture and biochemical properties. This is the case of K141E, K141N and many other disease-associated mutations occur within the conserved ACD domain, which is crucial for homodimer and heterodimer formation [28, 29, 37]. In addition, the K141N mutation may disrupt pathways that lead to fusion of the autophagosomes with lysosomes, since the K141 residue is required for binding to BAG3 [21]. Of note, a multiheteromeric complex composed of HspB8/BAG3/Hsc70/CHIP is responsible for directing misfolded proteins into an autophagosome [9].

*Drosophila melanogaster* has 11 predicted open-reading frames containing ACD domains. Several of them have been well characterized for their developmental and tissue-specific expression patterns, including Hsp27, 26, 23, and 22 [33, 35, 36]. Some of them revealed features related to their vertebrate orthologs. For example, *Drosophila* l [2] efl/dCryAB shares a role in the maintenance of muscle integrity with its vertebrate ortholog CRYAB (HSPB5) [53].

Since the Hsp67Bc sequence on its own does not immediately imply orthology to HSPB8, *Drosophila* Hsp67Bc was characterized as the functional ortholog of human HSPB8 interacting with Starvin, the sole BAG *Drosophila* protein [8]. Similar to HSPB8, HSP67Bc stimulates autophagy and reduces aggregation of ataxin-3 containing an expanded polyglutamine tract and a mutated form of HSPB1 that is associated with peripheral neuropathy. Moreover, Hsp67Bc, similar to its human counterpart, localizes to the Z-band in muscle tissue, suggesting that it could be a part of the conserved chaperone complex required for Z-disk maintenance [2, 8].

Here, we applied a *Drosophila* model to better understand the functional impact of pathogenic hot-spot HSPB8 mutations. Depending on the substitutions, we observed different abnormalities. Muscle-targeted expression of Hsp67BcR126E-Venus caused disruption of muscle structure reflected by the partial loss of the correct sarcomeric pattern, a decrease in the number of synaptic boutons per NMJ, and impairment of muscle performance. In turn, in Hsp67BcR126N-Venus muscles, we detected excessive accumulation of mutant proteins and the formation of large aggregates. Our data reveal that hot-spot residue substitutions in different *HSPB8* orthologs result in similar pathological muscle alterations.

## Materials and methods

### Drosophila husbandry

Fly stocks were raised on the standard corn meal-agar media. Fly crosses and experiments were carried out according to the standard procedures at 25 °C. The GAL4/UAS system was used to drive targeted gene expression. The following *Drosophila melanogaster* stocks were used: *w*^*1118*^, *Mef2*–*Gal4* (Bloomington Stock Center, 27390) and *UAS*-*GFP* (Bloomington Stock Center, 32201).

### Generation of UAS-Venus–Hsp67Bc, UAS-Venus–Hsp67BcR126E and UAS-Venus–Hsp67BcR126N lines

For plasmid construction, *Hsp67Bc*-coding sequences were amplified from cDNA obtained by reverse transcription of total RNA from the third instar larvae. PCR reaction using a high-fidelity DNA polymerase (Phusion, Biolabs) was performed with the pairs of primers listed below, containing the BamHI and XbaI restriction sites (see the table).

All amplified PCR products were digested with BamHI and XbaI and cloned into a pUASp-PL-Venus vector, which was then injected into *w*^*1118*^ embryos to produce transgenic flies, a step performed by the Fly-Facility platform (http://www.fly-facility.com Clermont-Ferrand, France).

To introduce R126E and R126N mutations into Hsp67Bc, we applied PCR-based mutagenesis. The PCR products carrying point mutations were generated with pairs of primers (see the table). Two microliters of each PCR product carrying a point mutation were mixed in 16 µl of water, heated to 99 °C, and annealed by cooling down to 37 °C within 25 min. Two microliters of annealed PCR products were then used for final PCR amplification with forward and reverse primers.

**Table:** 

Hsp67Bc-FOR	GGATCCGGATCCCCAGATATTCCCTTTGTCTTGAA
Hsp67Bc-REV	TCTAGATCTAGATCACTTGGCTTCTGGCTC
Hsp67Bc(R126N)-FOR	GCATTTTGTTAACCGCTATCCGCTGCC
Hsp67Bc(R126N)-REV	CAGCGGATAGCGGTTAACAAAATGCCG
Hsp67Bc(R126E)-FOR	GCATTTTGTTGAGCGCTATCCGCTGCC
Hsp67Bc(R126E)-REV	CAGCGGATAGCGCTCAACAAAATGCCG

All transgenic lines were P-element targeted insertions.

### Electrophoresis and Western blot

Larval brain lysates were analyzed by SDS-PAGE according to Laemmli using 12% mini-gels (Bio-Rad). For immunoblotting, after SDS-PAGE, the gels were electrophoretically transferred to nitrocellulose with transfer buffer (25 mM Tris, 192 mM glycine, 20% methanol, and 0.1% SDS) at room temperature (RT) for 1.5 h and at 25 V. The membrane with transferred proteins was blocked for 60 min at RT in blocking solution (5% nonfat dry milk in PBS with 0.05% Tween-20) and then incubated at RT overnight with rabbit polyclonal antibody raised against the C-terminal peptide CHKEAGPAASASEPEAK of *Drosophila melanogaster* HSP67Bc (Carra et al. [8]) diluted 1:5000. The membrane was then incubated at RT for 2 h with goat anti-rabbit IgG conjugated with horseradish peroxidase (Jackson ImmunoResearch) diluted 1:10,000 in PBST. Membranes were then detected and documented with a chemiluminescent method using the Bio-Rad Imaging System.

### Microscopy and immunohistochemistry

Third instar larvae were dissected in physiological salt with 25 mM EDTA. Body wall muscles were fixed with 4% formaldehyde in PBS for 15 min and then rinsed three times for 5 min each in PBS with 0.5% Tween 20 (PBT). Muscles were blocked for 30 min with 20% horse serum in PBT at RT. Staining was performed using primary antibodies applied overnight at 4 °C and after washing 3 times in PBT secondary antibodies were applied at RT for 1 h. The following antibodies were used: primary antibodies rabbit polyclonal antibody raised against the C-terminal peptide CHKEAGPAASASEPEAK of *Drosophila melanogaster* HSP67Bc [8] (a kind gift from Prof. Serena Carra), goat anti-GFP (1:500; Abcam, ab5450), rat anti-Kettin (1:25; Abcam, ab50585), mouse anti-MHC (1:1000, gift from D. Kiehart), mouse anti-LamC28.26 [1:1000; Developmental Studies Hybridoma Bank (DSHB)], anti-Brp1 (Bruchpilot—a marker of synaptic active zones) (1:100; DSHB, Nc82-s), anti-futsch/22C10 (1:100; DSHB, 22C10), anti-DLG (1:200; DSHB, 4F3 anti-disks large), and secondary antibodies goat anti-rat IgG conjugated to Alexa Fluor 488, CY3 or CY5 (1:300; Jackson ImmunoResearch).

For sarcomeric actin identification TRITC–phalloidin conjugated (1:1000; Sigma) or Alexa 546-conjugated phalloidin was used (at a concentration of 2 μg/ml; Life Technologies). DNA was stained with DAPI (4,6-diamidino-2-phenylindole; 0.2 μg/ml). The muscle was mounted in Fluoromount-G anti-fade reagent (Southern Biotech) or fluorescent mounting medium (Dako) and analyzed using an SP5 or SP8 (Leica) confocal microscope or an Olympus FluoView FV1000 confocal laser scanning microscope (Olympus). Any brightness and contrast adjustments were performed in FV10-ASW_Viewer or ImageJ.

### ImageJ-based corrected total cell fluorescence measurement

Evaluation of Venus-tagged Hsp67Bc variants’ expression levels was conducted using confocal images of ventral longitudinal VL4 muscles. The measurement of fluorescent signals was performed via the ImageJ-based corrected total cell fluorescence (CTCF) method [34]. Confocal images used for measurement were acquired with the same settings. The cytoplasmic aggregates present in Mef2 > Hsp67BcWT-Venus muscles were not quantified by the CTCF method.

Statistical analyses were carried out using Excel. Student’s *t* test was used for CTCF comparisons. The results are reported on the graph as the standard error of the mean and *P *< 0.05 is considered as statistically significant.

### Mitochondrial pattern in muscle fibers

The body wall preparations of the third instar larvae were stained with 500 nm MitoTracker Red CMXRos (Thermo Fisher) for 45 min. Tissues were then washed in 1 × PBS and fixed in 4% paraformaldehyde for 20 min. After a brief wash, they were mounted in 80% glycerol. For imaging, an Olympus FluoView FV1000 confocal laser scanning microscope (Olympus) was used. The images were recorded by employing Plan-Apochromat 10×, 20×, or 40× objectives. Any brightness and contrast adjustments were performed in FV10-ASW_Viewer or ImageJ.

### Transmission electron microscopy

The ultrastructural analysis was performed on a Zeiss EM 900 Transmission Electron Microscope. Dissected larvae were fixed in 2.5% glutaraldehyde in 0.1 M phosphate buffer, washed 3 times in phosphate buffer for 15 min, incubated overnight, and again washed 3 times. The material was post-fixed for 90 min in a mixture of osmium tetroxide and potassium ferricyanide in the ratio 1:1. After dehydration in an acetone series (50, 70, 90, 100, and 100%), the material was embedded in a mixture acetone–epoxy resin in the ratio 1:1 and incubated for 24 h in a dish. The dish was opened for at least 7 h for acetone to evaporate. Polymerization of material embedded in epoxy resin was performed during 24 h at 45 °C and subsequently for 3 days at 60 °C.

### Contractility and behavioral tests and *morphometric* characterization of the NMJ

Larval muscle performance was assessed using motility and contractility tests. Behavioral assays were carried out on 15 third instar larvae per genotype and repeated 3 times for each individual. In motility test, larvae were crawling on a Petri dish with 1% agar-juice medium and the number of peristaltic movements was counted during 30 s. The contractility test involves comparison of the length of muscle fibers relaxed by EDTA with that of contracted muscle fibers. The fiber contractility index (CI) was calculated from the following formula: CI = (size of relaxed fibers − size of contracted fibers)/size of relaxed fibers.

Morphometric characterization of the NMJ included calculation of the number of boutons, bouton diameter and number of branches per NMJ. It was conducted using longitudinal optical sections of lateral longitudinal LL1 surface area through synaptic boutons within the NMJ from Mef2 > Hsp67BcWT-Venus, Mef2 > Hsp67BcR126E-Venus, and Mef2 > Hsp67BcR126N-Venus larvae. The synaptic boutons were stained with anti-DLG antibodies.

Statistical analyses were carried out using Excel. Student’s *t* test and analysis of variance (ANOVA) were used for phenotype comparisons. The results are reported on the graphs as the standard error of the mean and *P *< 0.05 is considered as statistically significant.

## Results

### Drosophila melanogaster Hsp67Bc is a conserved sHSP

**Fig. 1:**
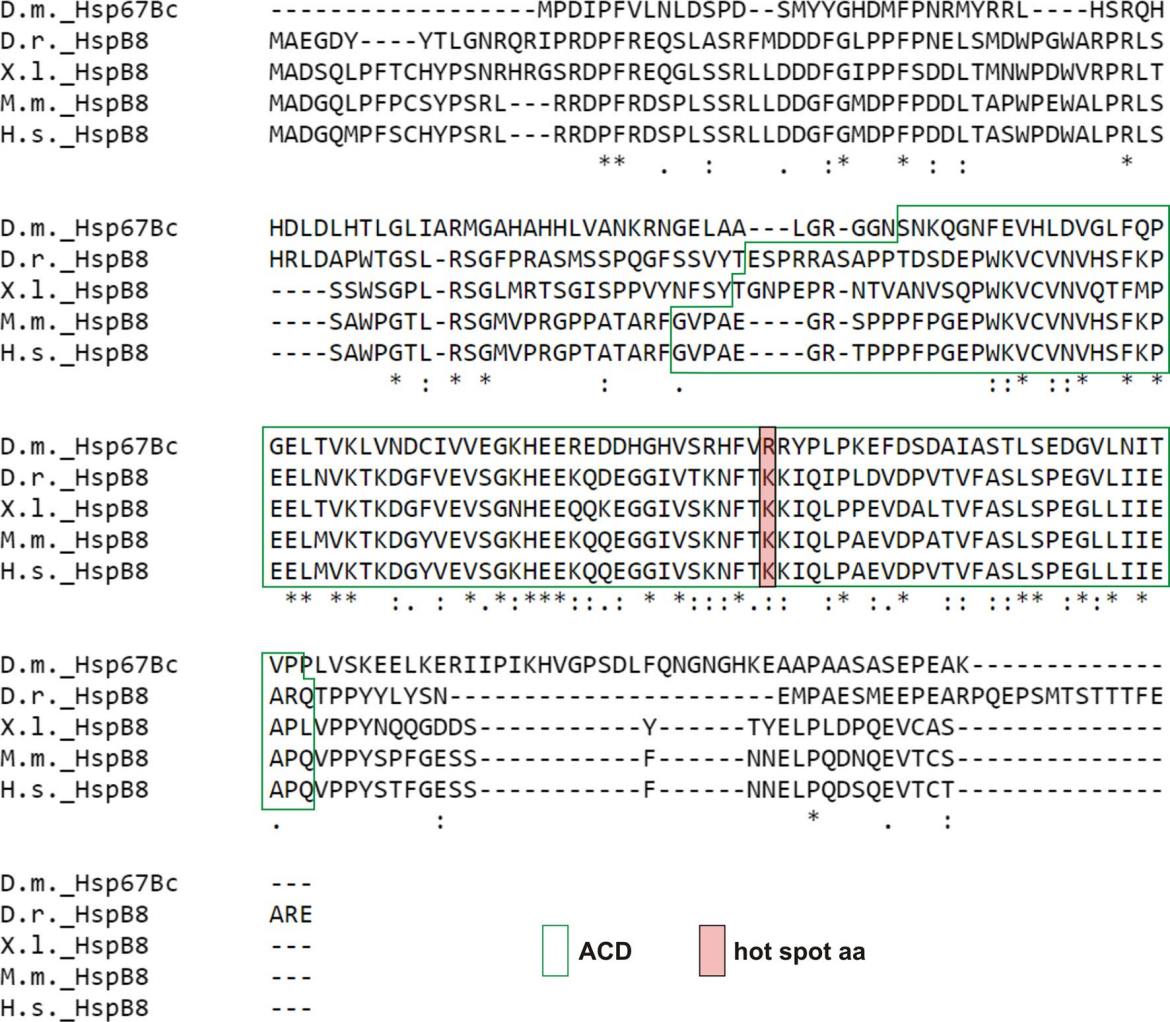
*Drosophila melanogaster* Hsp67Bc is a highly conserved member of the sHSP family and is related to the vertebrate HSPB8. Protein sequence alignment of *Drosophila melanogaster* (D.m._Hsp67BC), zebrafish (D.r._HSPB8), Xenopus laevis (X.l._HSPB8), mouse (M.m_HSPB8), and human HSPB8 (H.s._HSPB8) proteins. Conserved functional domain and amino acid residues (hot-spots) are highlighted by boxes: alpha-crystallin domain (ACD)—transparent with green frame, arginine 126 (R126) in *D. melanogaster* Hsp67Bc and lysine (K141) in human HSPB8—red with black frame. Consensus symbols in a sequence alignment have the following meaning: an asterisk (*) indicates a position which has a single, fully conserved residue, a colon (:) indicates conservation between groups of strongly similar properties, and a period (.) indicates conservation between groups of weakly similar properties. Sequence alignment: ClustalW2, http://www.ebi.ac.uk

**Fig. 2:**
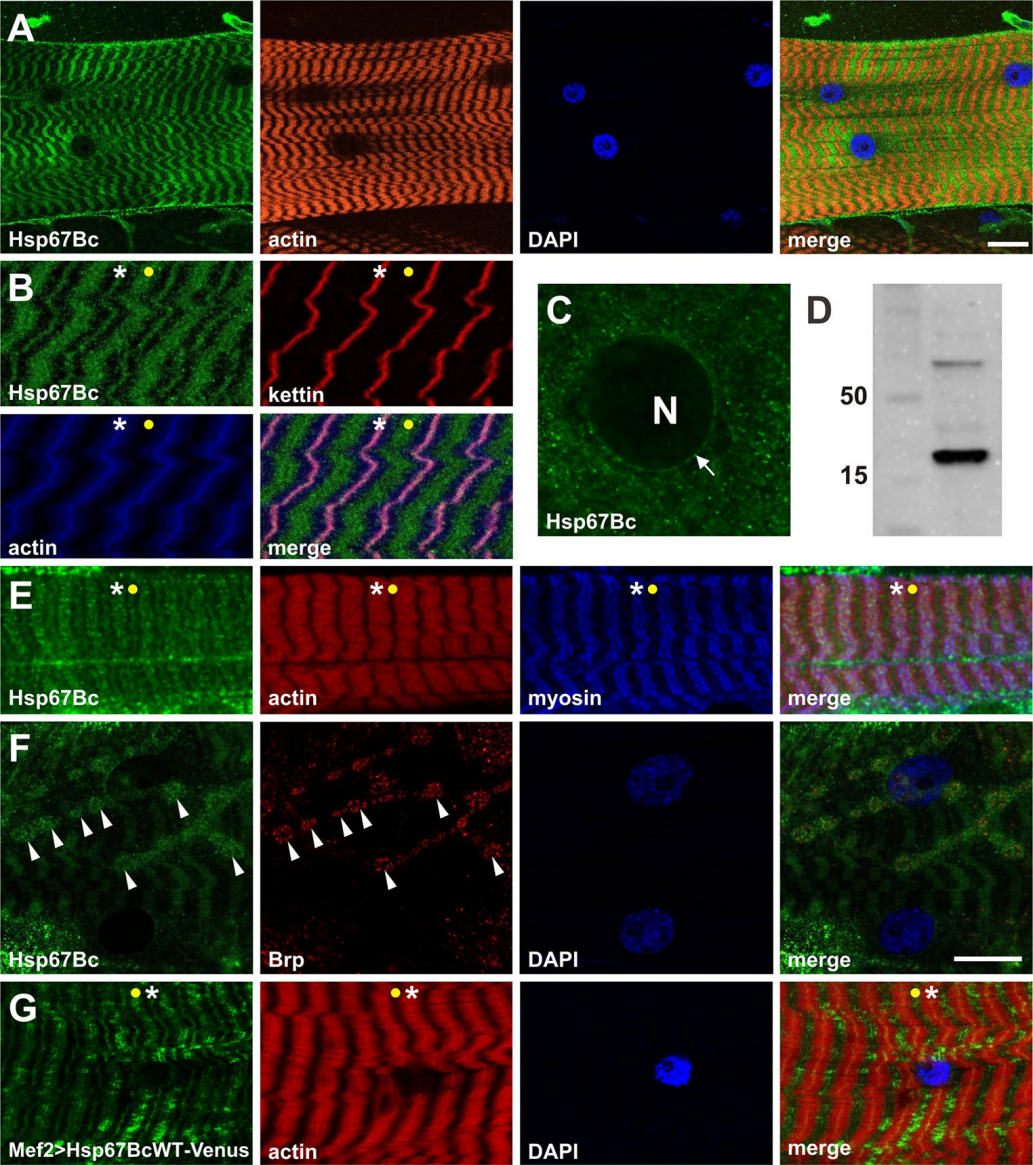
Expression pattern of Hsp67Bc in larval body wall muscles. **a** Hsp67Bc displays sarcoplasmic-associated expression in third instar larval muscles. Longitudinal optical sections of ventral longitudinal VL4. Anti-Hsp67Bc [8], TRITC–phalloidin for F-actin, DAPI. Scale bar: 15 μm. **b** Sarcomeric localization of Hsp67Bc. It is detected at the Z-bands (white asterisks) and in A-bands (yellow dot). Magnified longitudinal optical sections of ventral longitudinal VL4. Anti-Hsp67Bc [8], anti-kettin (DSHB), TRITC–phalloidin for F-actin, DAPI. **c** Hsp67Bc accumulation at the external surface of the nucleus (white arrow), N indicates nucleus. Magnified longitudinal optical sections of ventral longitudinal VL4. Anti-Hsp67Bc [8]. **d** Western blot of the third instar larvae protein extract showing the 22 kDa band of the expected size of the Hsp67Bc protein. **e** Sarcomeric localization of Hsp67Bc. Hsp67Bc is detected at the Z-bands (white asterisks) and in A-bands (yellow dot). Magnified longitudinal optical sections of ventral longitudinal VL4. Anti-Hsp67Bc [8], anti-MHC (gift from D. Kiehart),TRITC–phalloidin for F-actin, DAPI. **f** Sarcoplasmic distribution of Hsp67Bc. A small fraction of Hsp67Bc localizes at the NMJ (white arrowheads). Longitudinal optical sections of lateral longitudinal LL1. Anti-Hsp67Bc [8], anti-Brp1 (DSHB), DAPI. Scale bar: 15 μm. **g** Sarcomeric localization of Hsp67BcWT-Venus in Mef2 > Hsp67BcWT-Venus larvae. Like endogenous Hsp67Bc, it is detected at the Z-bands (white asterisks) and in A-bands (yellow dot). Local accumulations of overexpressed protein did not lead to destabilization of the myofibrillar organization. Magnified longitudinal optical sections of ventral longitudinal VL4. TRITC–phalloidin for F-actin, DAPI

The *Drosophila* Hsp67Bc (heat-shock protein 67Bc; CG4190) protein belongs to the family of small heat-shock proteins (sHSPs) and is thought to be the functional ortholog of vertebrate HSPB8 (Fig. 1) [8]. The *Hsp67B*c gene encodes a protein consisting of 199 amino acid residues with a predicted molecular mass of 22 kDa (Fig. 2d). The protein contains a conserved alpha-crystallin domain (ACD) comprising an immunoglobulin-like fold, which plays an important role in sHSP assembly. Moreover, approximately in the middle of the ACD domain, all HSPB8 orthologs carry an amino acid residue referred as a hot-spot (the most relevant residues for binding). In the human HSPB8, this residue corresponds to lysine 141 (K141) when mutated leads to pathological states such as neuropathy. In the case of *Drosophila* Hsp67Bc, a homologous amino acid residue, assigned by sequence alignment (Fig. 1), is arginine 126 (R126), which like lysine features positive side chains. Thus, based on interspecies sequence comparison, we hypothesized that Hsp67Bc R126 could represent a functional *Drosophila* hot-spot residue corresponding to HSPB8 K141.

### Stress-independent pattern expression of Hsp67Bc in larval body wall muscles

To examine the expression pattern of Hsp67Bc, we used previously described [8] rabbit polyclonal antibody raised against the C-terminal part of HSP67Bc. We found that Hsp67Bc is abundantly expressed in a stress-independent manner in the body wall muscles of the third instar larvae (Fig. 2a) and displays a striated sarcomeric pattern at the level of both the Z-line and the A-band (Fig. 2a, b, e asterisks and yellow dots). The granular pattern of Hsp67Bc expression in sarcomeres suggests that it might be part of large protein complexes. Moreover, we also detected perinuclear localization of Hsp67Bc in larval muscles (Fig. 2c, white arrow) as well as discrete muscle membrane associated expression restricted to the area of NMJs (Fig. 2f, white arrowheads).

Altogether, the observed Hsp67Bc expression pattern is reminiscent of that of its vertebrate orthologs [8], suggesting that it could play analogous functions in Z-disk stability and in maintenance of the NMJ.

We then tested whether expressing a Venus-tagged wild-type form of Hsp67Bc in larval muscles (using the Mef-Gal4 driver) could alter their structure. We first noticed that similar to endogenous Hsp67Bc (Fig. 2a, b), Hsp67Bc-Venus was detected at the Z-line (Fig. 2g, white asterisks) and A-band (Fig. 2g, yellow dot). We also found that this additional load of a wild-type form of Hsp67Bc in muscles did not affect the sarcomeric pattern (Fig. 2g). The Hspb67Bc-Venus expression pattern is reminiscent of that of endogenous Hsp67Bc. Moreover, we observed local accumulations of overexpressed protein along the muscle fibers, which did not lead to destabilization of the myofibrillar organization (Fig. 2g).

### Generation of pathogenic Hsp67Bc hot-spot variants and analyses of their impact on larval body wall muscles

To test whether R126 in *Drosophila* Hsp67Bc represents a hot-spot residue similar to K141 in human HSPB8, we generated inducible *Drosophila* transgenic lines carrying two different mutated forms of Hsp67Bc (R126E and R126N). To follow subcellular localization of these mutant variants, like for the wild-type Hsp67Bc, we incorporated a Venus tag. We then assessed whether *Drosophila* Hsp67Bc variants could mimic defects observed in patients with neuropathies and due to HSPB8 hot-spot mutations (K141E/N). We also investigated whether two different substitutions of the same amino acid residue could lead to distinct phenotypes. We decided to focus on muscle and thus, we chose tissue-specific induction under the Mef2–Gal4 driver.

**Fig. 3:**
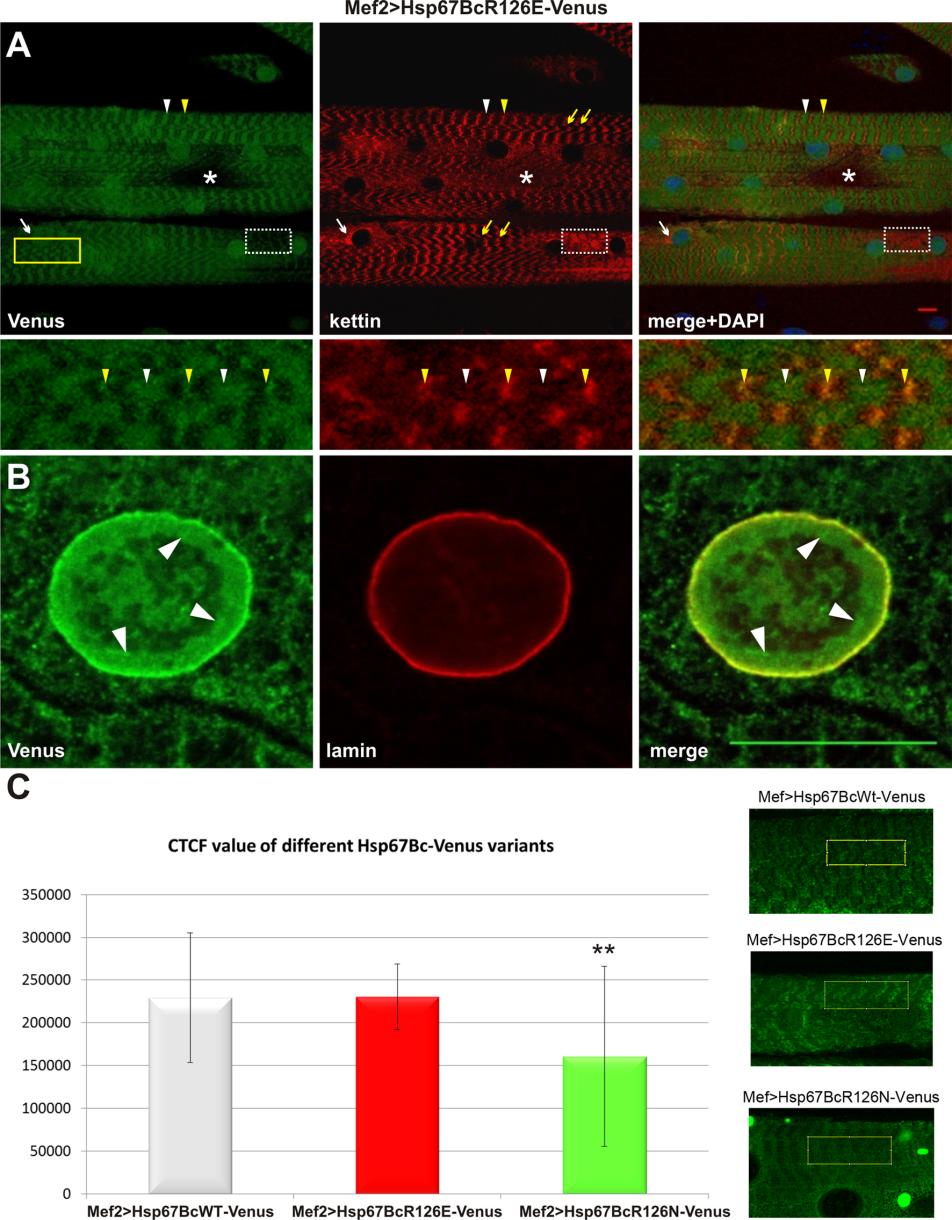
Phenotypic analysis of Mef2 > Hsp67BcR126E-Venus larval muscles. **a** Hsp67BcR126E-Venus displays accumulations along the muscle fibers retaining a striated sarcomeric pattern at the level of Z- (white arrowheads) and A-bands (yellow arrowheads). Note the presence of different local myofibrillar disruptions: Hsp67BcR126E-Venus perinuclear accumulation (white arrows), a significant reduction of Venus signal (dotted frame) accompanied by disturbances in kettin arrangement manifested by its local aggregation, complete loss of sarcomeric pattern (asterisks), and local misalignments of myofibrils (yellow arrows). Longitudinal optical sections of ventral longitudinal VL4. Anti-kettin (DSHB), DAPI. Scale bar: 10 μm. Inset: Magnification of region marked by yellow frame. Local misalignments of myofibrils. Hsp67BcR126E-Venus displays accumulations along the muscle fibers retaining a striated sarcomeric pattern at the level of Z- (white arrowheads) and A-bands (yellow arrowheads). **b** Translocation of a small fraction of the Hsp67BcR126E-Venus into the nucleus. The Hsp67BcR126E-Venus forms a relatively thick fraction in the proximity of the nuclear envelope (white arrowheads). Magnified longitudinal optical sections of ventral longitudinal VL4. Anti-LamC28.26 (DSHB), DAPI. Scale bar: 1 μm. **c** Evaluation of Venus-tagged Hsp67Bc variants’ expression levels in Mef2 > Hsp67BcWT-Venus, Mef2 > Hsp67BcR126E-Venus and Mef2 > Hsp67BcR126N-Venus ventral longitudinal VL4 muscles. The Image J based corrected total cell fluorescence (CTCF) method was applied for measurements of fluorescent signals. The cytoplasmic aggregates present in Mef2 > Hsp67BcR126N-Venus muscle were not quantified by the CTCF method. The Venus signal levels is comparable in Mef2 > Hsp67BcWT-Venus and Mef2 > Hsp67BcR126E-Venus muscles. The Venus signal in Mef2 > Hsp67BcR126N-Venus muscles is significantly lower in comparison with Mef2 > Hsp67BcWT-Venus. Error bars indicate SEM, *n* = 12 images per genotype; **P < 0.01 (Student’s *t* test). Inset: Exemplary confocal images of muscles expressing Venus-tagged Hsp67Bc variants with region marked by yellow frame used for signal measurements

**Fig. 4:**
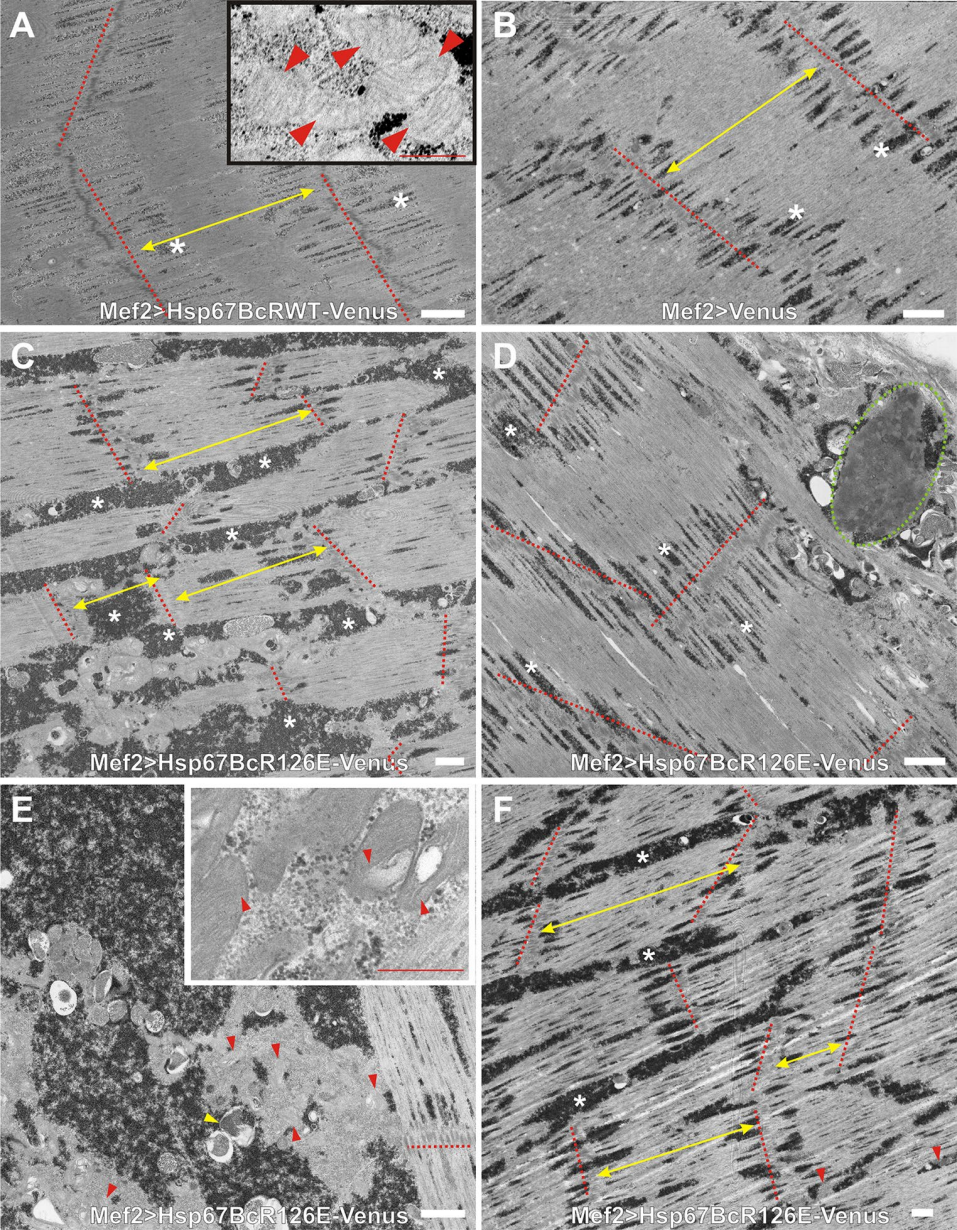
Ultrastructural analysis of Mef2 > Venus, Mef2 > Hsp67BcWT-Venus, and Mef2 > Hsp67BcR126E-Venus *D. melanogaster* larval muscles. **a** TEM analysis of Mef2 > Hsp67BcWT-Venus ventral longitudinal VL4 sarcomeres. Yellow double-ended arrows indicate regular distances between Z-lines traced by the red-dashed line. An asterisk indicates small deposits of glycogen granules, Scale bar: 1 μm. The inset shows muscle with functional mitochondria (red arrowheads). Scale bar: 0.4 μm. **b** TEM analysis of Mef2 > Venus ventral longitudinal VL4 sarcomeres. Yellow double-ended arrows indicate regular distances between Z-lines traced by red-dashed lines. An asterisk indicates small deposits of glycogen granules. Z-lines traced by red-dashed lines, and an asterisk indicates small deposits of glycogen granules. Scale bar: 1 μm. **c** TEM analysis of Mef2 > Hsp67BcR126E-Venus ventral longitudinal VL4 sarcomeres. Yellow double-ended arrows indicate interrupted and improperly placed Z-lines traced by the red-dashed lines. Asterisks indicate excessive accumulation of glycogen granules around the mitochondria and among sarcomeric units. Scale bar: 1 μm. **d** TEM analysis of Mef2 > Hsp67BcR126E-Venus ventral longitudinal VL4 sarcomeres. Asterisks indicate excessive accumulation of glycogen granules around the mitochondria and among sarcomeric units. Accumulation of disrupted mitochondria occurred in the subsarcolemmal sarcoplasm (encircled with green-dashed line). Scale bar: 1 μm. **e** TEM analysis of Mef2 > Hsp67BcR126E-Venus ventral longitudinal VL4 sarcomeres. Asterisks indicate excessive subsarcolemmal accumulation of glycogen granules around the mitochondria and among sarcomeric units, the membrane-bound autophagosomes containing glycogen (yellow arrowhead). Scale bar: 1 μm. The inset shows muscle with abrupt mitochondria characterized by the presence of broken cristae (red arrowheads). Scale bar: 0.4 μm. **f** TEM analysis of Mef2 > Hsp67BcR126E-Venus ventral longitudinal VL4 sarcomeres. Yellow double-ended arrows indicate interrupted and improperly placed Z-lines traced by the red-dashed lines. Asterisks indicate excessive accumulation of glycogen granules around the mitochondria (red arrowheads) and among sarcomeric units. Scale bar: 1 μm

To rule out the possibility that observed phenotypes are due to differences in expression levels of different Venus-tagged Hsp67Bc variants, we compared their expression levels using the ImageJ-based CTCF fluorescent signal measurement method [34] (Fig. 3c). We found a comparable level of transgene expression in muscles of both Mef2 > Hsp67BcWT-Venus and Mef2 > Hsp67BcR126E-Venus larvae (Fig. 3c). This indicates that phenotypes observed in the Mef2 > Hsp67BcR126E-Venus context (Figs. 3a, b; 4c–f, 7) can be ascribed to the presence of the introduced R126E mutation. The quantified fluorescent signal was significantly lower in the Mef2 > Hsp67BcR126N compared to the Mef2 > Hsp67BcWT larvae (Fig. 3c). This difference could be due to additional accumulation of the mutated (R126N) Hsp67Bc variant in cytoplasmic aggregates, which were not quantified by the CTCF measurements. However, we cannot entirely exclude that the observed minor muscle phenotype in Mef > Hsp67BcR126N context is related to R126N transgene expression level.

#### The R126E mutation leads to local sarcomeric pattern disruption affecting muscle performance and NMJ structure

The Hsp67BcR126E-Venus, similar to the endogenous Hsp67Bc (Fig. 2a, b) and Hsp67BcWT-Venus (Fig. 2g), revealed accumulations along the muscle fibers, retaining a striated sarcomeric pattern at the level of the Z-line and A-band (Fig. 3a, inset white and yellow arrowheads). However, this sarcomeric distribution was rather irregular, with local myofibrillar disruptions. Mef2 > Hsp67BcR126E-Venus muscle fibers displayed nuclear accumulation of the mutated variant (Fig. 3a, white arrows) and myofibrillar regions with a significant reduction (Fig. 3a, dotted frame) or complete loss of the sarcomeric Venus pattern (Fig. 3a, asterisks). Interestingly, the significant reduction of the Venus signal, which correlates with the distribution of the examined mutant protein, was accompanied by an affected kettin arrangement manifested by its local aggregation (Fig. 3a, dotted frame). In addition, we occasionally observed local misalignments of myofibrils (Fig. 3a, yellow arrows, yellow frame, inset), which may indicate local destabilization of myofibrillar organization caused by Hsp67BcR126E-Venus overexpression.

One unexpected change in Mef2 > Hsp67BcR126E-Venus was the translocation of a small fraction of the Hsp67BcR126E-Venus into nuclei (Fig. 3b). Instead of being restricted to a very thin layer surrounding the external surface of the nucleus (Fig. 2c), the mutated Hsp67BcR126E-Venus variant occupied the internal nucleus area, forming a relatively thick fraction in the proximity of the nuclear envelope (Fig. 3b, white arrowheads).

To better characterize the influence of Hsp67BcR126E-Venus, we decided to conduct an ultrastructural analysis of *Drosophila* larval muscles expressing this mutant protein (Fig. 4c–f). Transmission electron microscopy (TEM) analysis of the different parts of individual muscle fibers confirmed our confocal laser scanning microscopy (CLSM) observations and revealed the presence of interrupted and improperly spaced Z-lines (Fig. 4c, f, red-dashed lines, double-ended yellow arrows). We also observed excessive accumulation of glycogen granules [32] around the mitochondria and among sarcomeric units (Fig. 4c, e, f, white asterisks), as well as the membrane-bound autophagosomes containing glycogen (Fig. 4e, yellow arrowhead) compared to Mef2 > Venus and Mef2 > Hsp67BcWT-Venus larval muscles (Fig. 4a, b). Moreover, mitochondria were characterized by the presence of broken cristae (Fig. 4e, inset, red arrowheads), which suggested their impaired respiratory function. TEM analysis demonstrated that in Mef2 > Hsp67BcR126E-Venus muscles, accumulation of disrupted mitochondria occurred in the subsarcolemmal sarcoplasm (Fig. 4d, encircled with green-dashed line).

Since muscle organization in Mef2 > Hsp67BcR126E-Venus larvae in both CLSM and TEM appeared to be significantly altered in comparison with wild-type individuals, we decided to check whether the presence of mutated protein affected larval muscle function (Fig. 7a, b, red bars). To test muscle performance, we used crawling and muscle contractility tests. We found that Mef2 > Hsp67BcR126E-Venus larvae revealed reduced muscle function (Fig. 7a, b, red bars) compared to Mef2 > Venus (Fig. 7a, b, black bars) and Mef2 > Hsp67BcWT-Venus (Fig. 7a, b, gray bars) individuals.

We additionally assessed the mitochondrial function using MitoTracker staining, whose accumulation is dependent upon membrane potential (Fig. 7c). We observed a reduction of the mitochondrial signal (Fig. 7c), indicating abrupt respiratory function. This result is in line with TEM analysis, which revealed the presence of abnormal mitochondria with broken cristae and numerous glycogen deposits in Mef2 > Hsp67BcR126E-Venus muscles (Fig. 4e).

We also tested whether the presence of the Hsp67BcR126E hot-spot mutant affects the NMJ structure. Morphometric characterization of the NMJ indicated a decreased number of synaptic boutons (Fig. 7d, red bar, Fig. 7g) in Mef2 > Hsp67BcR126E-Venus larvae but no changes in bouton diameter or the number of branches per NMJ (Fig. 7e, f, red bars, Fig. 7g).

#### The R126N mutation leads to large aggregate formation and disturbs the sarcomeric pattern without affecting muscle performance

**Fig. 5:**
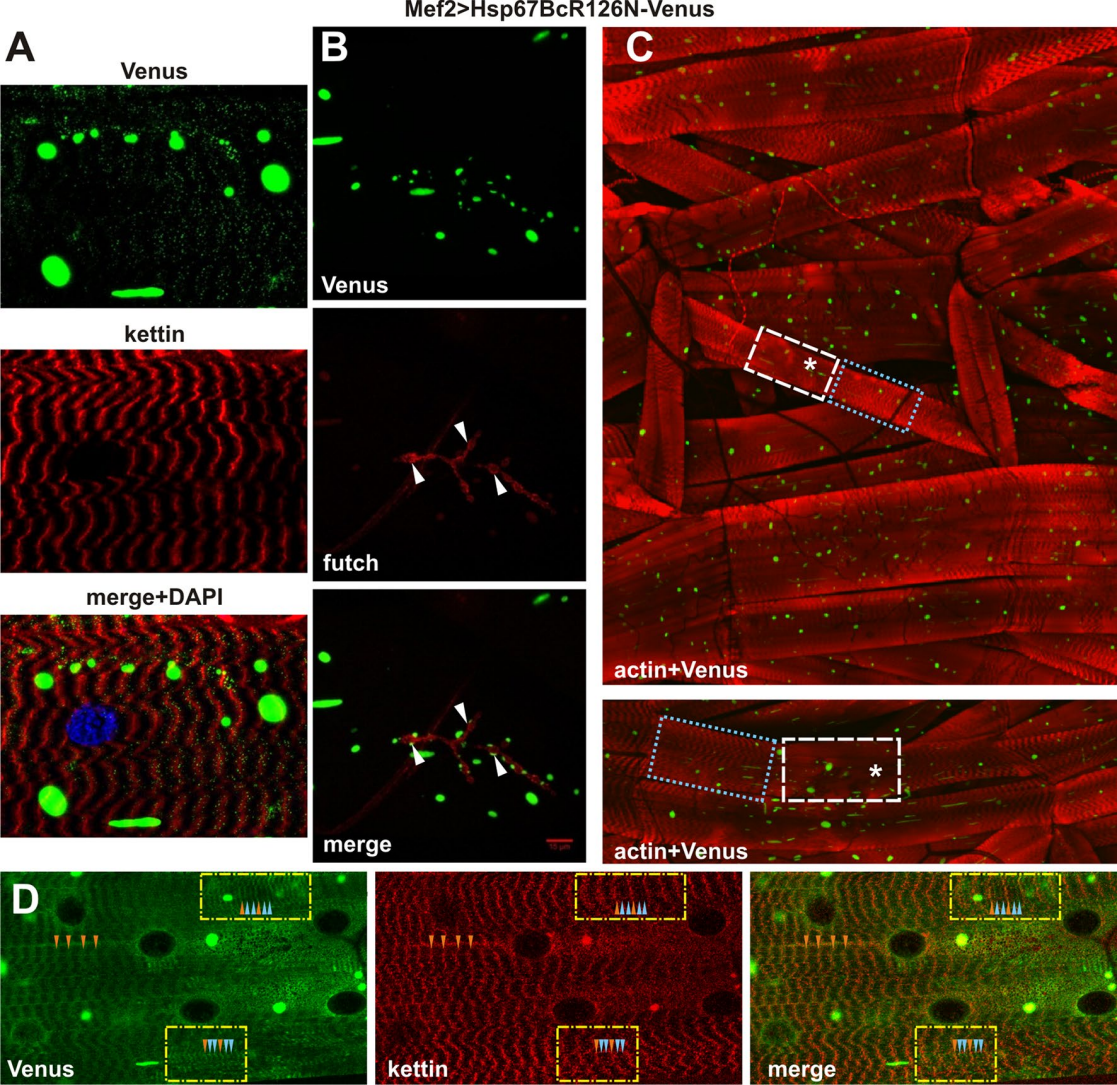
Phenotypic analysis of Mef2 > Hsp67BcR126N-Venus larval muscles. **a** Hsp67BcR126N-Venus distribution is disrupted to a significantly lesser extent. Longitudinal optical sections of ventral longitudinal VL4. Note the presence of large green aggregates. Anti-kettin (DSHB), DAPI. **b** The aggregate-prone form of Hsp67BcR126N-Venus retained its ability to localize at the NMJ sites (white arrowheads). Note the presence of large green aggregates. Anti-futsch/22C10 (DSHB). Scale bar: 15 μm. **c** Hsp67BcR126N-Venus distribution in ventral part of hemisegment musculature. Besides regions which appeared normal with regularly interspaced Z-bands (blue dotted box), we observed neighboring regions with attenuated intensity of actin bands (asterisks) and regions with a condensed sarcomeric pattern (white dashed box). Note the presence of large green aggregates. Inset in the bottom shows magnified longitudinal optical sections of ventral longitudinals VL4 and VL3. 3D reconstruction of confocal scans through ventral part of hemisegment musculature (Olympus FV1000 software). TRITC–phalloidin for F-actin. **d** Magnified longitudinal optical sections of ventral longitudinal VL4. Hsp67BcR126N-Venus localized in additional stripes seen as two bands (blue arrowheads) on each side of the Z-line (orange arrowheads). Note the presence of large green aggregates. Anti-kettin (DSHB), DAPI

Compared to Hsp67BcWT-Venus (Fig. 2g), the muscle-targeted expression of the second hot-spot Hsp67BcR126N-Venus variant led to an irregular sarcomeric pattern along the muscle fibers (Fig. 5d) with a significantly lower sarcomere-associated Venus signal. However, compared to Mef2 > Hsp67BcR126E-Venus (Fig. 3a), Mef2 > Hsp67BcR126N-Venus larvae showed no nuclear localization of the mutant protein and generally less severe alterations of the sarcomeric pattern (Fig. 5a, c).

Besides numerous and large regions which appeared normal with regularly interspaced Z-bands (Fig. 5c, blue dotted box), we noted neighboring regions with attenuated intensity of actin bands (Fig. 5c, asterisks) and regions with a condensed sarcomeric pattern (Fig. 5c, white dashed box) and a high phalloidin signal. Occasionally, we observed that Hsp67BcR126N-Venus was localized in additional stripes seen as two bands (Fig. 5d, blue arrowheads) on each side of the Z-line (Fig. 5d, orange arrowheads). We also observed areas of accumulated Hsp67BcR126N-Venus (Fig. 5d) in which the sarcomeric architecture was not particularly affected.

**Fig. 6:**
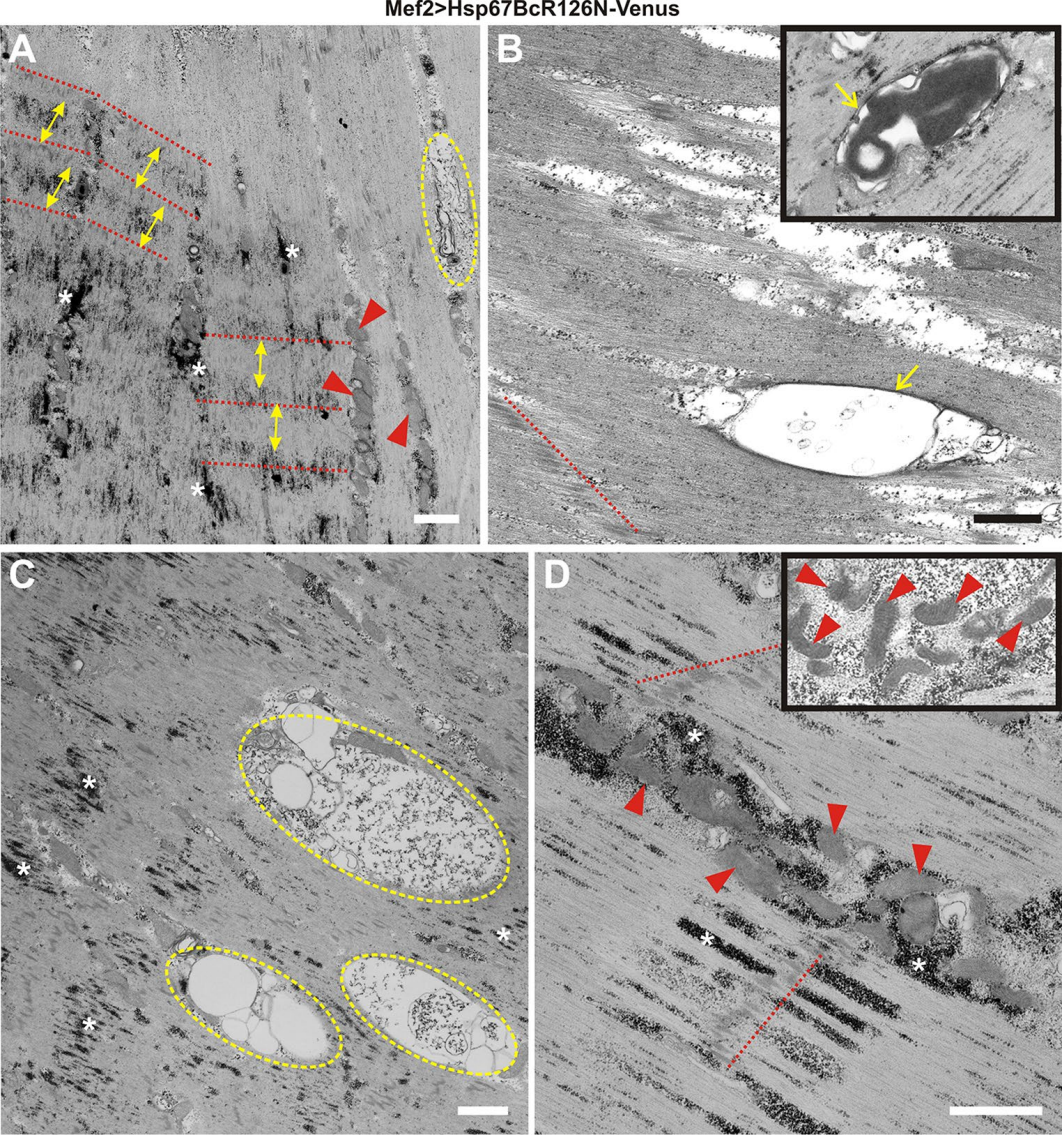
Ultrastructural analysis of *D. melanogaster* Mef2 > Hsp67BcR126N-Venus larval muscles. **a** TEM analysis of Mef2 > Hsp67BcR126N-Venus ventral longitudinal VL4 sarcomeres. The condensed sarcomeric pattern observed results from local compaction of sarcomeres. Yellow double-ended arrows indicate regular distances between Z-lines traced by the red-dashed lines. An asterisk indicates deposits of glycogen granules. Muscles reveal normal mitochondria (red arrowheads) distribution. Note the presence of membrane-bound autophagosome encompassing amorphous aggregate (encircled with dashed yellow line). Scale bar: 1 μm. **b** TEM analysis of Mef2 > Hsp67BcR126N-Venus ventral longitudinal VL4 sarcomeres. Membrane-bound (yellow arrow) autophagosome encompassing amorphous aggregate localized along sarcomeric unit. Z-line traced by red-dashed line. Inset shows membrane-bound (yellow arrow) autophagosome encompassing electron-dense aggregate. Scale bar: 1 μm. **c** TEM analysis of Mef2 > Hsp67BcR126N-Venus ventral longitudinal VL4 sarcomeres. Large and numerous membrane-bound autophagosomes encompassing amorphous aggregate (encircled with dashed yellow line) are localized within sarcomeric units. Asterisk indicates deposits of glycogen granules. Scale bar: 1 μm. **d** TEM analysis of Mef2 > Hsp67BcR126N-Venus ventral longitudinal VL4 sarcomeres. Muscles revealed normal mitochondria (red arrowheads) distribution. Z-lines traced by red-dashed lines. An asterisk indicates deposits of glycogen granules. Inset shows muscle with functional mitochondria (red arrowheads) with properly formed cristae. Scale bar: 1 μm

To further investigate the local destabilization of myofibrillar organization in Mef2 > Hsp67BcR126N-Venus muscles, we performed ultrastructural analyses (Fig. 6a–d). The condensed sarcomeric pattern observed in the confocal microscope (Fig. 5c) corresponds with local compaction of sarcomeres, whereas the attenuated intensity of actin bands may be related to local loss of sarcomeres (Fig. 6a). Of note, in Mef2 > Hsp67BcR126N-Venus larvae, sarcomeres were interspaced more regularly compared to Mef2 > Hsp67BcR126E-Venus muscles (Fig. 4c, f). In addition, TEM analysis of Mef2 > Hsp67BcR126N-Venus muscle revealed normal mitochondria distribution (Fig. 6a, d, red arrowheads). The mitochondria had proper organization of cristae (Fig. 6d, inset, red arrowheads).

The most spectacular effect of the second substitution, R126N, was the formation of large aggregates with different contents in muscle fibers (Figs. 5a–d, 6a–c). Taking into account the localization, shape, and size of the aggregates displaying a strong Venus signal (Fig. 5a–d), we concluded that they correspond to structures observed in TEM (Fig. 6a–c, encircled with dashed yellow line). Moreover, the ultrastructural analysis revealed the presence of membrane-bound (Fig. 6b, yellow arrows, c) autophagosomes containing amorphous aggregate and electron-dense cytosolic components.

We also analyzed the distribution of Hsp67BcR126N-Venus at the NMJ (Fig. 5b) and compared it to the distribution of the endogenous Hsp67Bc (Fig. 2f). Intriguingly, an aggregate-prone form of Hsp67BcR126N-Venus retained its ability to localize at the NMJ sites (Fig. 5b, white arrowheads), forming aggregates in the vicinity of the NMJ.

**Fig. 7:**
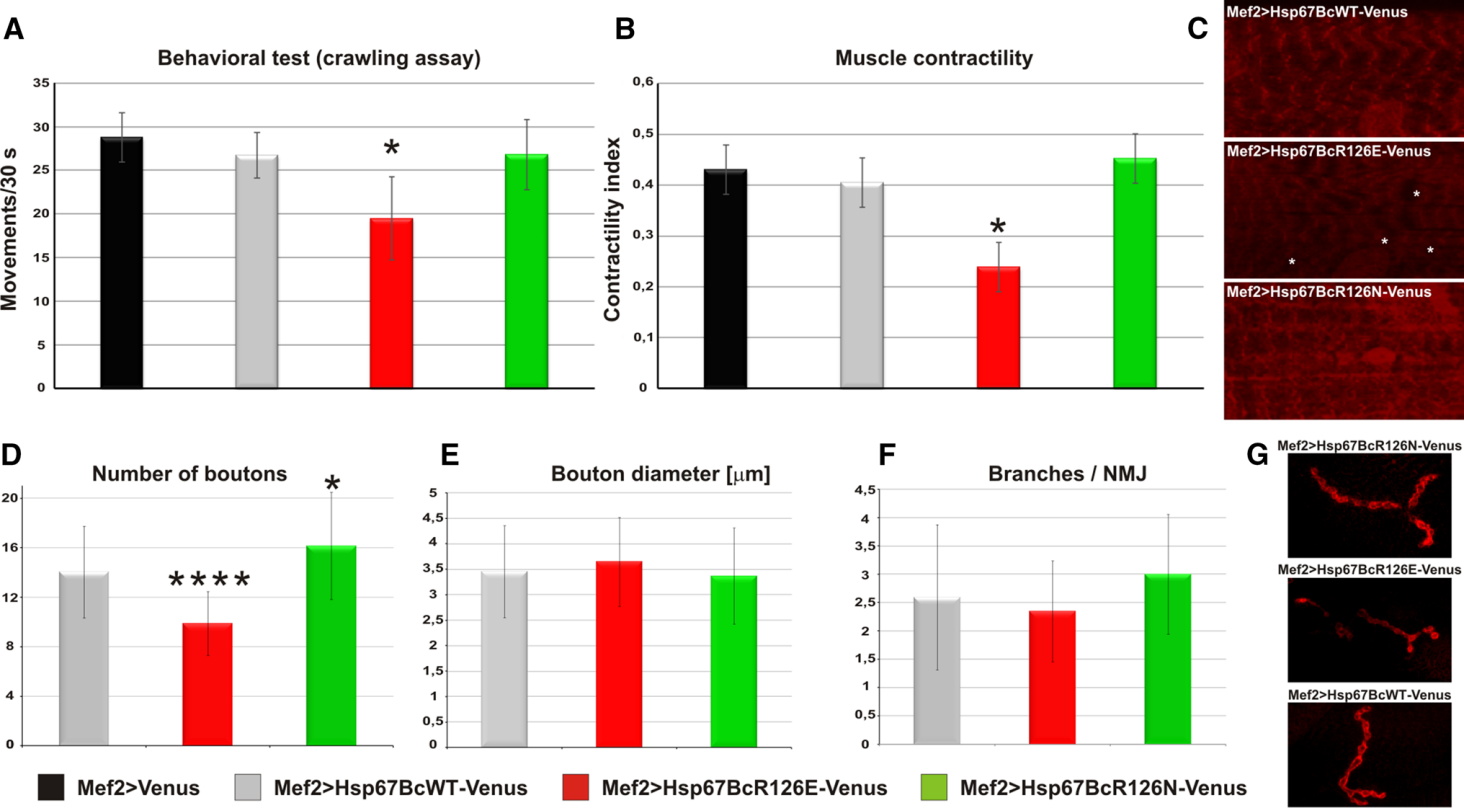
Evaluation of muscle performance and NMJ morphology in Mef2 > Hsp67BcWT-Venus, Mef2 > Hsp67BcR126E-Venus and Mef2 > Hsp67BcR126N-Venus third instar larvae. **a** Muscle crawling test shows reduced motility of Mef2 > Hsp67BcR126E-Venus larvae (red bars) in comparison with control Mef2 > Venus larvae (black bars), Mef2 > Hsp67BcWT-Venus larvae (gray bars), and Mef2 > Hsp67BcR126N-Venus larvae (green bars). Error bars indicate SEM (standard error of mean). Tests were repeated 3 times for each individual, *n* = 15 flies per genotype, *P < 0.05 (Student’s *t* test/ANOVA). **b** Muscle contractility test shows affected contractility of Mef2 > Hsp67BcR126E-Venus larvae (red bars) in comparison with control Mef2 > Venus larvae (black bars), Mef2 > Hsp67BcWT-Venus larvae (gray bars), and Mef2 > Hsp67BcR126N-Venus larvae (green bars). The fiber contractility index (CI) was calculated from the following formula: CI = (size of relaxed fibers − size of contracted fibers)/size of relaxed fibers. Error bars indicate SEM, *n* = 15 flies per genotype; *P < 0.05 (Student’s *t* test/ANOVA). **c** Muscle staining with MitoTracker Red CMXRos (Thermo Fisher), whose accumulation is dependent upon membrane potential. We observed a reduction of the mitochondrial signal, indicating an abrupt respiratory function in the case of Mef2 > Hsp67BcR126E-Venus larvae compared to Mef2 > Hsp67BcWT-Venus or Mef2 > Hsp67BcR126N-Venus larvae. An asterisk indicates a region containing reduction of the mitochondrial signal. Magnified longitudinal optical sections of ventral longitudinal VL4. **d** NMJ (neuromuscular junction) formation is affected in Mef2 > Hsp67BcR126E-Venus and Mef2 > Hsp67BcR126N-Venus larvae, which is reflected in the abnormal number of boutons. The muscle-target expression of Hsp67BcR126E-Venus significantly reduces the number of boutons formed during development (indicated by four asterisks), whereas the presence of Hsp67BcR126N-Venus affects it to a lesser extent, causing an increase of the number of boutons (indicated by one asterisk). Error bars indicate SEM, *n* = 30 NMJs per genotype; *P < 0.05, ****P < 0.00001 (Student’s *t* test/ANOVA). **e** The muscle-targeted expression of Hsp67BcR126E-Venus and Hsp67BcR126N-Venus does not affect bouton diameter. Error bars indicate SEM, *n* = 30 NMJs per genotype; *P < 0.05 (Student’s *t* test/ANOVA). **f** The muscle-targeted expression of Hsp67BcR126E-Venus and Hsp67BcR126N-Venus does not affect the number of branches per NMJ. Error bars indicate SEM, *n* = 30 NMJs per genotype; *P < 0.05 (Student’s *t* test/ANOVA). **g** Magnified longitudinal optical sections of lateral longitudinal LL1 surface area from Mef2 > Hsp67BcWT-Venus, Mef2 > Hsp67BcR126E-Venus or Mef2 > Hsp67BcR126N-Venus larvae. 3D reconstruction of confocal scans through synaptic boutons within the NMJ (Olympus FV1000 software). Anti-DLG (DSHB)

As in the case of the Mef2 > Hsp67BcR126E-Venus, the alterations observed in Mef2 > Hsp67BcR126N-Venus muscle prompted us to test whether this mutated form also affected larval muscle function. Thus, we tested muscle performance using contractility and motility tests (Fig. 7a, b). Surprisingly, muscle activity of Mef2 > Hsp67BcR126N-Venus larvae (Fig. 7a, b, green bars) did not differ statistically significantly from either Mef2 > Hsp67BcWT-Venus (Fig. 7a, b, gray bars) or Mef2 > Venus individuals (Fig. 7a, b, black bars). Accordingly, we did not observe a reduction of the mitochondrial signal (Fig. 7c), indicating that respiratory functions are not affected in Mef2 > Hsp67BcR126N-Venus muscles.

In addition, assessment of the NMJ morphology in the Mef2 > Hsp67BcR126N-Venus individuals revealed that the second hot-spot mutation has no impact on the NMJ structure (Fig. 7d–f, green bars, Fig. 7g).

## Discussion

All sHSP class members have many common features, with respect to both structure and function, independently of the species they come from. Structural similarity includes the presence of an ACD carrying a residue with a positive charge (usually lysine, arginine, or histidine) referred to as a hot-spot residue [20]. These highly conserved amino acids are crucial for proper oligomerization and chaperone activity [6]. Their substitutions may bring dramatic changes resulting in the development of various pathological states.

Hsp67Bc, similar to human HSPB8, localizes to sarcomere Z-bands. Due to direct interaction with Starvin, a counterpart of Bag3, Hsp67Bc contributes to autophagosome-driven protein degradation [8]. The previous studies showed that in *Drosophila* CryAB (human HSPB5) also localizes at the Z-band [53]. Our experiments confirmed Z-band localization of Hsp67Bc and revealed for the first time its presence in the sarcomere A-band. The presence of Hsp67Bc in sarcomere structures suggests that like CryAB, it could control sarcomere stability.

Here, we applied a *Drosophila* model to test the impacts of hot-spot mutations in the gene coding for HSPB8 that have been associated with two inherited human pathologies, the autosomal dominant dHMN (K141E) [27], and CMT disease type 2L (K141N) [47]. In those cases, the positively charged residue is substituted either with glutamic acid (E), which has acidic side chains at neutral pH, or with asparagine (N), which has a polar but not charged side chain. The ACD encoded by Hsp67Bc, the *Drosophila* HSPB8 ortholog, carries an analogous hot-spot residue (R126), which has been mutated to generate pathological variants (R126E and R126N). We observed that when targeted to developing muscles, these two mutations of the same residue result, as in humans, in different structural and functional defects. Both disease-mimicking R126E and R126N mutated forms of Hsp67Bc showed changes in their distribution pattern and affected larval muscle structure. Muscle-specific expression of Hsp67BcR126N-Venus led to a significantly greater extent of aggregate formation in comparison with Hsp67BcR126E-Venus overexpression and to a local loss of sarcomeric pattern. This observation suggests that *Drosophila* hot-spot Hsp67BcR126N mutants could serve as a model for studying protein aggregation-related diseases.

In contrast, at the ultrastructural level more dramatic changes were observed in the Hsp67BcR126E mutant context. Despite glycogen accumulation being present in both cases, the Hsp67BcR126E mutation leads to abrupt sarcomeric units with interrupted and irregularly spaced Z-bands, revealing that Hsp67Bc, similar to its vertebrate counterpart, is involved in the stabilization of Z-disks. Moreover, in the Hsp67BcR126E context mitochondria were characterized by the presence of broken cristae and the MitoTracker signal was significantly reduced, suggesting impaired respiratory function. In addition, examination of the synapse structure showed a decrease in the number of synaptic boutons per NMJ. All these structural muscle defects could have contributed to the reduced larval muscle performance and inefficient muscle contraction observed in Mef2 > Hsp67BcR126E-Venus larvae.

The impact of the R126N mutation on the PQC system manifested by increased formation of cytoplasmic aggregates, as well as the presence of the R126E-mutated form in perinuclear aggregates, supports the idea of Hsp67Bc involvement in protein sorting and lysosome-mediated degradation. Moreover, because in Mef2 > Hsp67BcR126N-Venus mutants, some of the membrane-bound structures present in muscles resembled aggresomes, we hypothesize that their presence ensures the maintenance of muscle homeostasis reflected in undisturbed muscle performance. Indeed, aggresome formation plays a beneficial role ensuring sequestration of aggregated, and therefore toxic proteins, which left in their soluble oligomeric form could be more harmful to cellular processes [49].

Although we did not observe lethality in Mef2 > Hsp67BcR126E-Venus or Mef2 > Hsp67BcR126N-Venus larvae, the effects of both mutations support their dominant toxic gain of function. Further analyses are required to correlate generated fly models with late onset dHMNII and CMT2L muscular diseases.

Interestingly, the similarities of phenotypes obtained by Hsp67Bc and CryAB hot-spot mutant overexpression may be connected with the fact that both proteins (HSPB8 and HSPB5) have been shown to form heterodimers with each other [19, 46, 53]. It is worth noting that enhanced autophagosome formation, which we detected in Hsp67BcR126N muscles, was also observed in hearts of mice with CryAB^R120G^ [48]. However, as suggested by studies conducted on a slightly different CryAB^R120G^ mouse model in which autophagic activity was decreased, autophagosome formation does not necessarily reflect autophagic activity [31].

On the other hand, K141E and K141N mutant variants could have distinct functional impacts. For example, in the case of HEK-293T (human embryonal kidney) cells, the ability of the mutated K141N form to protect the cell against aggregation was significantly decreased, whereas the mutated K141E form showed an almost complete loss of its protective function [8]. It has also been reported that both hot-spot mutations lead to significant reduction of neuronal cell viability [27] and neurite degeneration reflected in their reduced number and length [25]. Moreover, cells from a dHMNII patient revealed the presence of HSPB8 aggregates, reduction of mitochondrial membrane potential [26] and defects in HSPB8-mediated autophagy [9–12, 41]. Furthermore, it was reported that HSPB8^K141N^ in motor neuron-like cells (NSC34, neuroblastoma–spinal cord hybrid cells) can initiate autophagy but cannot bring it to completion. The authors suggested that autophagy impairment occurs during lysosomal delivery or fusion rather than after the substrate recognition and autophagosome formation [30]. Importantly, impairment of autophagy and the UPS is involved in the pathogenesis of muscle disorders [40]. Differentiated tissues such as skeletal muscles are susceptible to destroyed organelles and aggregate-prone proteins whose presence may significantly affect cellular homeostasis, leading to contractile apparatus destabilization and muscle weakness. The detailed dissection of the pathway underlying pathological conditions in muscle described here might help to develop therapeutic treatment of diseases.

Notably, mutations in other members of the sHSP family such as HSPB1 and HSPB5 have also been associated with pathological states, whose etiology is underlined by protein aggregation [13, 18, 23, 24, 50, 53]. In the case of HSPB5, substitution of arginine localized at a conserved position in the ACD with glycine (R120G) led to desmin-related myofibrillar myopathy (DRM) manifested by alteration of the spatial organization of the mutated protein [13, 23, 24, 53]. Other molecular effects of this mutation include formation of aggregates containing desmin in muscle fibers, irregular sarcomeric architecture, and abnormal mitochondrial organization [23, 39, 50, 53].

The purpose of our study was to characterize the tissue-specific influence of Hsp67Bc mutants (R126E and R126N) on muscle structure and on the morphology of NMJs. We also analyzed the impact of Hsp67Bc mutations on muscle function and found that both the aberrant sarcomeric organization and an abnormal number of synaptic buttons within NMJs could contribute to affected mutants’ mobility. The neuromuscular synaptic context of impaired action of sHSPs was previously suggested [45]. Moreover, a mutation in HSPB1 interferes with essential neuronal processes, e.g., axonal transport [1, 18], and the defects in BAG3/HSPB8-mediated autophagy proved to be pathologic in neural cells [42]. This is in line with the observation revealing localization of sHSPs in the vicinity of synapses [4]. We analyzed the distribution of Hsp67BcR126N-Venus at the muscular side of the NMJ and compared it to the distribution of the endogenous Hsp67Bc. Intriguingly, an aggregate-prone form of Hsp67BcR126N-Venus retained its ability to localize at the NMJ sites, but its localization differed significantly from the distribution of the endogenous form and had no impact on the NMJ structure.

In the light of these data, it would be worthwhile to test the influence of Hsp67Bc hot-spot mutations on neural function. A comparison with data presented here would bring new insights into Hsp67Bc’s tissue-specific roles and its disease-connected mutation.

## Abbreviations

CryAB: B-crystallin
PQC: Protein quality control
l(2)efl: Lethal (2) essential for life
BAG3: Bcl-2-associated athanogene-3
dHMN: Distal hereditary motor neuropathy
CMT: Charcot–Marie–Tooth disease
NMJ: Neuromuscular junction

## References

[1] Ackerley S, James PA, Kalli A, French S, Davies KE, Talbot K (2006). A mutation in the small heat-shock protein HSPB1 leading to distal hereditary motor neuronopathy disrupts neurofilament assembly and the axonal transport of specific cellular cargoes. Hum Mol Genet.

[2] Arndt V, Dick N, Tawo R, Dreiseidler M, Wenzel D, Hesse M, Fürst DO, Saftig P, Saint R, Fleischmann BK, Hoch M, Höhfeld J (2010). Chaperone-assisted selective autophagy is essential for muscle maintenance. Curr Biol.

[3] Bakthisaran R, Akula KK, Tangirala R (1860). Rao ChM (2016) Phosphorylation of αB-crystallin: role in stress, aging and patho-physiological conditions. Biochim Biophys Acta.

[4] Bechtold DA, Brown IR (2000). Heat-shock proteins Hsp27 and Hsp32 localize to synaptic sites in the rat cerebellum following hyperthermia. Brain Res Mol Brain Res.

[5] Benndorf R, Sun X, Gilmont RR, Biederman KJ, Molloy MP, Goodmurphy CW, Cheng H, Andrews PC, Welsh MJ (2001). HSP22, a new member of the small heat shock protein superfamily, interacts with mimic of phosphorylated HSP27 (3DHSP27). J Biol Chem.

[6] Bera S, Thampi P, Cho WJ, Abraham EC (2002). A positive charge preservation at position 116 of alpha A-crystallin is critical for its structural and functional integrity. Biochemistry.

[7] Carra S (2009). The stress-inducible HspB8–Bag3 complex induces the eIF2alpha kinase pathway: implications for protein quality control and viral factory degradation?. Autophagy.

[8] Carra S, Boncoraglio A, Kanon B, Brunsting JF, Minoia M, Rana A, Vos MJ, Seidel K, Sibon OC, Kampinga HH (2010). Identification of the *Drosophila* ortholog of HSPB8: implication of HSPB8 loss of function in protein folding diseases. J Biol Chem.

[9] Carra S, Crippa V, Rusmini P, Boncoraglio A, Minoia M, Giorgetti E, Kampinga HH, Poletti A (2012). Alteration of protein folding and degradation in motor neuron diseases: Implications and protective functions of small heat shock proteins. Prog Neurobiol.

[10] Carra S, Seguin SJ, Landry J (2008). HspB8 and Bag3: a new chaperone complex targeting misfolded proteins to macroautophagy. Autophagy.

[11] Crippa V, Carra S, Rusmini P, Sau D, Bolzoni E, Bendotti C, De Biasi S, Polett A (2010). A role of small heat shock protein B8 (HspB8) in the autophagic removal of misfolded proteins responsible for neurodegenerative diseases. Autophagy.

[12] Crippa V, Sau D, Rusmini P, Boncoraglio A, Onesto E, Bolzoni E, Galbiati M, Fontana E, Marino M, Carra S, Bendotti C, De Biasi S, Poletti A (2010). The small heat shock protein B8 (HspB8) promotes autophagic removal of misfolded proteins involved in amyotrophic lateral sclerosis (ALS). Hum Mol Genet.

[13] Dalakas MC, Park KY, Semino-Mora C, Lee HS, Sivakumar K, Goldfarb LG (2000). Desmin myopathy, a skeletal myopathy with cardiomyopathy caused by mutations in the desmin gene. N Engl J Med.

[14] Dierick I, Irobi J, De Jonghe P, Timmerman V (2005). Small heat shock proteins in inherited peripheral neuropathies. Ann Med.

[15] Dubińska-Magiera M, Jabłońska J, Saczko J, Kulbacka J, Jagla T, Daczewska M (2014). Contribution of small heat shock proteins to muscle development and function. FEBS Lett.

[16] Duennwald ML, Echeverria A, Shorter J (2012). Small heat shock proteins potentiate amyloid dissolution by protein disaggregases from yeast and humans. PLoS Biol.

[17] Ecroyd H, Meehan S, Horwitz J, Aquilina JA, Benesch JLP, Robinson CV, Macphee CE, Carver JA (2007). Mimicking phosphorylation of aB-crystallin affects its chaperone activity. Biochem J..

[18] Evgrafov OV, Mersiyanova I, Irobi J, Van Den Bosch L, Dierick I, Leung CL, Schagina O, Verpoorten N, Van Impe K, Fedotov V, Dadali E, Auer-Grumbach M, Windpassinger C, Wagner K, Mitrovic Z, Hilton-Jones D, Talbot K, Martin JJ, Vasserman N, Tverskaya S, Polyakov A, Liem RK, Gettemans J, Robberecht W, De Jonghe P, Timmerman V (2004). Mutant small heat-shock protein 27 causes axonal Charcot–Marie–Tooth disease and distal hereditary motor neuropathy. Nat Genet.

[19] Fontaine JM, Sun X, Hoppe AD, Simon S, Vicart P, Welsh MJ, Benndorf R (2006). Abnormal small heat shock protein interactions involving neuropathy-associated HSP22 (HSPB8) mutants. FASEB J..

[20] Franck E, Madsen O, van Rheede T, Ricard G, Huynen MA, de Jong WW (2004). Evolutionary diversity of vertebrate small heat shock proteins. J Mol Evol.

[21] Fuchs M, Poirier DJ, Seguin SJ, Lambert H, Carra S, Charette SJ, Landry J (2010). Identification of the key structural motifs involved in HspB8/HspB6–Bag3 interaction. Biochem. J..

[22] Ghosh JG, Estrada MR, Clark JI (2005). Interactive domains for chaperone activity in the small heat shock protein, human alphaB crystallin. Biochemistry.

[23] Goldfarb LG, Dalakas MC (2009). Tragedy in a heartbeat: malfunctioning desmin causes skeletal and cardiac muscle disease. J Clin Invest..

[24] Goldfarb LG, Vicart P, Goebel HH, Dalakas MC (2004). Desmin myopathy. Brain.

[25] Irobi J, Almeida-Souza L, Asselbergh B, De Winter V, Goethals S, Dierick I, Krishnan J, TimmermansJP Robberecht W, De Jonghe P, Van Den Bosch L, Janssens S, Timmerman V (2010). Mutant HSPB8causes motor neuron-specific neurite degeneration. Hum Mol Genet.

[26] Irobi J, Holmgren A, De Winter V, Asselbergh B, Gettemans J, Adriaensen D, Ceuterick-de Groote C, VanCoster R, De Jonghe P, Timmerman V (2012). Mutant HSPB8 causes protein aggregates and a reducedmitochondrial membrane potential in dermal fibroblasts from distal hereditary motor neuropathy patients. Neuromuscul Disord.

[27] Irobi J, Van Impe K, Seeman P, Jordanova A, Dierick I, Verpoorten N, Michalik A, De Vriendt E, Jacobs A, Van Gerwen V, Vennekens K, Mazanec R, Tournev I, Hilton-Jones D, Talbot K, Kremensky I, Van Den Bosch L, Robberecht W, Van Vandekerckhove J, Van Broeckhoven C, Gettemans J, De Jonghe P, Timmerman V (2004). Hot-spot residue in small heat-shock protein 22 causes distal motor neuropathy. Nat Genet.

[28] Kasakov AS, Bukach OV, Seit-Nebi AS, Marston SB, Gusev NB (2007). Effect of mutations in the beta5-beta7 loop on the structure and properties of human small heat shock protein HSP22 (HspB8, H11). FEBS J.

[29] Kim MV, Kasakov AS, Seit-Nebi AS, Marston SB, Gusev NB (2006). Structure and properties of K141E mutant of small heat shock protein HSP22 (HspB8, H11) that is expressed in human neuromuscular disorders. Arch Biochem Biophys.

[30] Kwok AS, Phadwal K, Turner BJ, Oliver PL, Raw A, Simon AK, Talbot K, Agashe VR (2011). HspB8 mutation causing hereditary distal motor neuropathy impairs lysosomal delivery of autophagosomes. J Neurochem.

[31] Maloyan A, Sayegh J, Osinska H, Chua BH, Robbins J (2010). Manipulation of death pathways in desmin-related cardiomyopathy. Circ Res.

[32] Marchand I, Chorneyko K, Tarnopolsky M, Hamilton S, Shearer J, Potvin J, Graham TE (2002). Quantification of subcellular glycogen in resting human muscle: granule size, number, and location. J Appl Physiol.

[33] Marin R, Tanguay RM (1996). Stage-specific localization of the small heat shock protein Hsp27 during oogenesis in *Drosophila melanogaster*. Chromosoma.

[34] McCloy RA, Rogers S, Caldon CE, Lorca T, Castro A, Burgess A (2014). Partial inhibition of Cdk1 in G 2 phase overrides the SAC and decouples mitotic events. Cell Cycle.

[35] Michaud S, Tanguay RM (2003). Expression of the Hsp23 chaperone during *Drosophila* embryogenesis: association to distinct neural and glial lineages. BMC Dev Biol.

[36] Morrow G, Tanguay RM (2003). Heat shock proteins and aging *in Drosophila melanogaster*. Semin Cell Dev Biol.

[37] Mymrikov EV, Bukach OV, Seit-Nebi AS, Gusev NB (2010). The pivotal role of the beta 7 strand in the intersubunit contacts of different human small heat shock proteins. Cell Stress Chaperones.

[38] Mymrikov EV, Seit-Nebi AS, Gusev NB (2011). Large potentials of small heat shock proteins. Physiol Rev.

[39] Perng MD, Wen SF, van den IJssel P, Prescott AR, Quinlan RA (2004). Desmin aggregate formation by R120G αB-crystallin is caused by altered filament interactions and is dependent upon network status in cells. Mol Biol Cell.

[40] Sandri M, Coletto L, Grumati P, Bonaldo P (2013). Misregulation of autophagy and protein degradation systems in myopathies and muscular dystrophies. J Cell Sci.

[41] Sau D, Rusmini P, Crippa V, Onesto E, Bolzoni E, Ratti A, Poletti A (2011). Dysregulation of axonal transport and motorneuron diseases. Biol Cell.

[42] Seidel K, Vinet J, Dunnen WF, Brunt ER, Meister M, Boncoraglio A, Zijlstra MP, Boddeke HW, Rüb U, Kampinga HH, Carra S (2012). The HSPB8-BAG3 chaperone complex is upregulated in astrocytes in the human brain affected by protein aggregation diseases. Neuropathol Appl Neurobiol.

[43] Selcen D, Muntoni F, Burton BK, Pegoraro E, Sewry P, Bite AV, Engel AG (2009). Mutation in BAG3 causes severe dominant childhood muscular dystrophy. Ann Neurol.

[44] Sreelakshmi Y, Santhoshkumar P, Bhattacharyya J, Sharma KK (2004). AlphaA-crystallin interacting regions in the small heat shock protein, alphaB-crystallin. Biochemistry.

[45] Stetler RA, Gan Y, Zhang W, Liou AK, Gao Y, Cao G, Chen J (2010). Heat shock proteins: cellular and molecular mechanisms in the CNS. Prog Neurobiol.

[46] Sun X, Fontaine J, Rest J, Shelden E, Welsh M, Benndorf R (2004). Interaction of human HSP22 (HSPB8) with other small heat shock proteins. J Biol Chem.

[47] Tang BS, Zhao GH, Luo W, Xia K, Cai F, Pan Q, Zhang RX, Zhang FF, Liu XM, Chen B, Zhang C, Shen L, Jiang H, Long ZG, Dai HP (2005). Small heat-shock protein 22 mutated in autosomal dominant Charcot–Marie–Tooth disease type 2L. Hum Genet.

[48] Tannous P, Zhu H, Johnstone JL, Shelton JM, Rajasekaran NS, Benjamin IJ, Nguyen L, Gerard RD, Levine B, Rothermel BA, Hill JA (2008). Autophagy is an adaptive response in desmin-related cardiomyopathy. Proc Natl Acad Sci USA.

[49] Tyedmers J, Mogk A, Bukau B (2010). Cellular strategies for controlling protein aggregation. Nat Rev Mol Cell Biol.

[50] Vicart P, Caron A, Guicheney P, Li Z, Prévost MC, Faure A, Chateau D, Chapon F, Tomé F, Dupret JM, Paulin D, Fardeau M (1998). A missense mutation in the alphaB-crystallin chaperone gene causes a desmin-related myopathy. Nat Genet.

[51] Vos MJ, Hageman J, Carra S, Kampinga HH (2008). Structural and functional diversities between members of the human HSPB, HSPH, HSPA, and DNAJ chaperone families. Biochemistry.

[52] Wang X, Osinska H, Klevitsky R, Gerdes AM, Nieman M, Lorenz J, Hewett T, Robbins J (2001). Expression of R120G-alphaB-crystallin causes aberrant desmin and alphaB-crystallin aggregation and cardiomyopathy in mice. Circ Res.

[53] Wójtowicz I, Jabłońska J, Zmojdzian M, Taghli-Lamallem O, Renaud Y, Junion G, Daczewska M, Huelsmann S, Jagla K, Jagla T (2015). *Drosophila* small heat shock protein CryAB ensures structural integrity of developing muscles, and proper muscle and heart performance. Development.

